# Who is at risk for alcohol-related negative consequences?

**DOI:** 10.1186/1940-0640-7-S1-A42

**Published:** 2012-10-09

**Authors:** Paola Pedrelli, Charlotte Brill, Fava Maurizio

**Affiliations:** 1 Department of Psychology, Massachusetts General Hospital/Harvard Medical School, Boston, MA, USA; 2 Depression Clinical & Research Program, Massachusetts General Hospital, Boston, MA, USA

## 

Although a large body of research has documented that severe consequences are associated with alcohol use among college students, this may not be the case for all students who consume alcohol. Identifying those variables associated with risk for negative consequences in this population has important public health implications. Mixed findings are available on the relationship between alcohol-related negative consequences and alcohol consumed, depressive symptoms, and use of illicit substances. We examined the relationship between alcohol-related consequences and amount consumed during a typical and a heavy drinking week, cannabis use in the previous 12 months, and depressive symptoms. Participants consisted of a convenience sample of 85 (53.5% females) undergraduate college students (mean age = 19 years) taking part in an on-campus mental-health screening. Participants reported use of alcohol and of other drugs, mental heath symptoms, and negative consequences. Results showed a direct association between alcohol-related negative consequences and total drinks consumed during a typical week (r = 0.41; p < 0.001) and during a heavy drinking week (r = 0.58; p < 0.001). Students with any cannabis use in the previous 12 months reported a significant greater number of alcohol-related negative consequences (t [1, 80] = -2.66; p < 0.01). Alcohol-related negative consequences were not associated with depressive symptoms. In a hierarchical linear regression model including cannabis use, total drinks during a typical week, and total drinks during a heavy drinking week, the latter was the only predictor of alcohol-related negative consequences. The other two variables did not predict any variance over and above the one explained by total drinks during a heavy drinking week. In conclusion, programs aimed at reducing the negative outcomes of alcohol consumption should aim at screening for heavy rather than typical drinking.

